# Surgical treatment and outcome of sterile prostatic cysts in dogs

**DOI:** 10.1111/vsu.13642

**Published:** 2021-05-07

**Authors:** Sara Del Magno, Guido Pisani, Francesco Dondi, Filippo Cinti, Emanuela Morello, Marina Martano, Armando Foglia, Davide Giacobino, Paolo Buracco

**Affiliations:** ^1^ Department of Veterinary Medical Sciences, Alma Mater Studiorum University of Bologna Bologna Italy; ^2^ Centro Veterinario Luni Mare La Spezia Italy; ^3^ Eastcott Referrals Veterinary Hospital Swindon UK; ^4^ Department of Veterinary Science University of Turin Turin Italy

## Abstract

**Objective:**

To describe the surgical treatment and outcome of a large cohort of dogs with sterile prostatic cysts (PCs).

**Study Design:**

Retrospective study.

**Animals:**

Forty‐four client‐owned dogs.

**Methods:**

Dogs with sterile PCs with at least 6 months of follow‐up were included. Clinical variables, type of surgery, complications, recurrences, and outcomes (telephonic interviews or rechecks) were recorded.

**Results:**

Extra‐ and intraparenchymal cysts were diagnosed in 29 and 11 dogs, respectively. Four dogs had both types. Extraparenchymal cysts were treated by partial resection and omentalization (*n* = 22) and complete resection (*n* = 7). Drainage and intracapsular omentalization were performed in all dogs with intraparenchymal cysts. The four dogs with both types of cyst were treated by omentalization. Resolution was documented in 39/44 dogs (88.6%). Intraoperative complications occurred in one dog (urethral tear). Major complications resulting in death occurred in three dogs (oliguric kidney injury, cardiac arrhythmia, and persisting urinary tract obstruction). Minor complications (*n* = 10) consisted of temporary urinary incontinence (*n* = 2), permanent urinary incontinence (*n* = 5), urinary retention (*n* = 2), and dysuria (*n* = 1). Recurrence occurred in two dogs with extraparenchymal cysts. Median long‐term follow‐up was 528 days (range, 250–730 days). Thirty‐nine dogs had no signs associated with prostatic disease at long‐term follow‐up.

**Conclusion:**

Partial or complete resection and/or omentalization of sterile PCs led to resolution of clinical signs in most dogs, although postoperative urinary incontinence was frequent.

**Impact:**

This study is the largest case series relative to canine sterile PCs treated surgically and provides evidence on the prognosis and rate of complications.

## 1 INTRODUCTION

Prostatic diseases are relatively common in older, intact male, large‐breed dogs. Benign prostatic hyperplasia (BPH) and prostatitis are frequently diagnosed while prostatic cysts (PCs) (including both intra‐ and extraparenchymal cysts), abscesses, neoplasia, and metaplasia are infrequently reported.^1^, ^2^, ^3^, ^4^ In particular, PCs have an incidence of approximately 5% of diagnosed prostatic diseases,^2^ and can become infected in up to 42% of cases.^3^

Intraparenchymal cysts (previously called retention cysts) are believed to originate from microcysts, with an accumulation of prostatic gland secretions due to increased production or obstruction of the ducts during BPH.^5^, ^6^ They can also contain urine and have a connection with the urethra.^7^, ^8^ Extraparenchymal cysts (previously called paraprostatic cysts) have a connection with the prostatic capsule, but they do not involve the prostatic parenchyma.^5^ They are thought to be associated with the Müllerian duct remnant,^6^ although this finding has never been proven.

Clinical signs associated with PCs are frequently the result of compression on the adjacent organs of the caudal abdomen or inside the pelvic canal which may result in dysuria, urinary retention, dyschezia, and ribbon‐like feces.^5^ Hematuria is common in many prostatic disorders and urinary incontinence has also been reported.^5^ In case of secondary infection of the cyst, systemic illness can be observed.^5^, ^9^ Abdominal enlargement can be present and, on rectal examination and/or caudal abdominal palpation, enlargement of the gland is frequently detected.^5^ Ultrasound examination usually confirms the clinical suspicion.^1^ Perineal hernia has also been reported concurrently with PCs and BPH.^5^, ^10^, ^11^

In veterinary medicine, the treatment of PCs has changed in recent decades, involving either surgical or non‐surgical procedures. Cyst aspiration,^8^, ^12^ alcoholization,^13^ and injection of platelet‐rich plasma^14^ have been proposed as an alternative to surgery; however, clear guidelines regarding patient selection are lacking.^5^ Historical surgical procedures such as the application of passive drainage, marsupialization, or partial prostatectomy are still listed as possible treatments; however, they are no longer recommended.^5^, ^15^, ^16^ More recently, surgical procedures, such as partial cyst wall resection and extracapsular omentalization, or drainage with intracapsular omentalization, have been proposed.^5^, ^10^, ^17^ At present, surgery for PCs is recommended as a definitive treatment or in cases of recurrence after the use of minimally invasive procedures.^5^ Only one case series has described the surgical management of PC in 18 dogs followed during a medium to long‐term follow‐up^17^; most of the remaining veterinary literature includes studies dealing with only small numbers of dogs followed for a short period of time (range from no follow‐up to 24 months, with only 7 dogs followed for more than 5 months).^7^, ^8^, ^10^, ^18^

The aim of the present study was to describe the surgical treatment, follow‐up, and outcome in a large cohort of dogs with sterile PCs.

## 2 MATERIALS AND METHODS

The medical records of dogs with prostatic disease at the Ospedale Universitario Veterinario, University of Turin, and Centro Veterinario Luni Mare in the period 1998–2017 were retrospectively reviewed.

Dogs were included in the study if they underwent surgical treatment of sterile (based on bacterial culture and cytology) PCs. Cyst classification was based on its position relative to the prostate gland according to the diagnostic imaging and surgical findings. Specifically, when the cyst was surrounded and intimately connected to the prostatic parenchyma, it was considered to be intraparenchymal; if the cyst was only connected to the prostatic capsule, it was considered to be extraparenchymal. Moreover, only dogs with PCs treated by complete cyst resection, partial resection^5^ and omentalization,^17^ or drainage and intracapsular omentalization^19^ were enrolled in the study. A minimum follow‐up of 6 months was required for inclusion in the study, with the exception of those dogs that died or developed major complication before this period.

The anesthetic protocol was chosen by the anesthesiologist on duty; however, in general, after sedation and anesthesia induction, the dogs underwent inhalation anesthesia with isoflurane (IsoFlo, Zoetis Italia srl, Milano, Italy) in a mixture of oxygen and air following orotracheal intubation. All the dogs received cephazolin (Cefazolina TEVA, Teva Italia srl, Milano, Italy) at anesthesia induction (22 mg/kg IV), which was repeated every 90 min during surgery. Intraoperative analgesia varied according to the anesthesiologist's preference.

The dogs were positioned in dorsal recumbency and had the hair clipped over the abdominal and inguinal region. In case of extension of the cyst into the perineum or of a concomitant perineal hernia, hair clipping was extended to the perineum, and a purse‐string suture was placed around the anus with non‐absorbable monofilament material. The prepuce, abdomen, and perineum underwent aseptic preparation before surgery. A polyethylene urethral catheter of a diameter adequate to the dog's size was placed and connected to a closed‐collection system. After orchiectomy, for PC located in the caudal abdomen, a caudal midline celiotomy was performed, the abdomen was carefully inspected, and the cystic lesions were isolated from the surrounding organs with laparotomy gauze. In case of extension of the PCs in the pelvic canal, an additional separate perineal approach was also performed. A perineal approach was performed as a single approach for PCs located in the pelvic canal and perineal region only.

For intraparenchymal cysts, surgery consisted of opening the prostatic capsule in an area overlying the cyst, and drainage of the fluid. In case of multiple cysts divided by septa, these were broken either digitally or using mosquito forceps in an attempt to create a single cavity; the urethra was identified by palpation of the urethral catheter. The cavity was then irrigated, suctioned, and finally omentalized. The unfolded ventral leaf of the omentum was carefully dissected from the dorsal leaf closed to its attachment to the greater curvature of the stomach and was loosely wrapped around the urethra, with the help of mosquito forceps, being careful not to damage the urethra.^19^ The omentum was sutured over itself with some tacking sutures using 3/0 absorbable monofilament material.

For extraparenchymal cysts, drainage of the cystic fluid content was carried out by means of either a 22 gauge needle connected to a 20‐ or 50‐ml syringe or a Poole suction tip (connected to an aspiration device); the suction tip was inserted through a stab incision in the cyst wall and fixed with a purse‐string suture applied just before the stab incision. After drainage of the cystic content, a complete excision, or partial resection and omentalization were performed with the unfolded omentum, as described earlier. In the latter case, most of the cystic wall was removed, trying to avoid damage to the dorsolateral part of the bladder neck, and the remaining cystic tissue was irrigated with saline. The omentum was sutured to the inside of the remaining cyst wall or the prostatic capsule with 3/0 absorbable monofilament material.^17^

In case of concomitant presence of intra‐ and extraparenchymal cysts, both types of cysts were treated by omentalization, as described for every single type of cyst.

Postoperative antimicrobial therapy was administered to those dogs for whom no cytological examination or bacterial culture was available before surgery. It consisted of amoxycillin and clavulanic acid (Synulox, Zoetis, Rome, Italy), 20 mg/kg every 12 h orally, or marbofloxacin (Marbocyl, Vetoquinol, Magny‐Vernois, France), 3 mg/kg every 24 h orally for 7 postoperative days. Analgesia with opioids (buprenorphine 0.015 mg/kg every 6 h IM or methadone 0.1 mg/kg every 4–6 h IM) and intravenous fluids were administered. Meloxicam (Boehringer Ingelheim, Ingelheim, Germany) 0.1 mg/kg every 24 h orally was administered to all dogs except those with altered renal values. In cases where the surgeon had doubts about the integrity of the urethra, or when preoperative dysuria was present, the polypropylene urinary catheter was exchanged for a Foley catheter that was left in place during the hospitalization period. Bloodwork was repeated in the case of preoperative abnormalities.

Data regarding age, breed, findings on clinical examination, diagnostic imaging performed, comorbidities, and surgical treatment were analyzed; intraoperative and postoperative complications were recorded.

Postoperative complications were considered minor if they were self‐limiting or when medical treatment was able to resolve the problem. Major postoperative complications were defined as life‐threatening conditions or those requiring surgical intervention. Recurrence of the PCs was also recorded and, in the case of revision surgery, only the time to recurrence was considered. Deaths (including euthanasia) related to surgery during the first postoperative month were recorded. The long‐term follow‐up of the dogs was carried out by evaluating the medical records in the electronic database, or by telephone interview with the referring veterinarian.

### 2.1 Statistical analysis

Data were analyzed using descriptive statistics and reported as median and range (minimum–maximum value).

## 3 RESULTS

Seventy‐five dogs with surgically treated, non‐neoplastic prostatic disease were identified. Of these 75 dogs, 73 had cavitated lesions and 2 had squamous metaplasia. An abscess or an infected PC was diagnosed in 26 dogs which were consequently excluded from the study, leaving 47 dogs with a sterile PC. Three of the earlier dogs were treated with marsupialization and were thus excluded from the study population. Therefore, 44 dogs met the inclusion criteria and were included in the study.

### 3.1 Demographics

All the 44 dogs were intact males, and 2/44 were unilateral cryptorchid with the scrotal testis already removed before presentation. The median age was 8 years (range, 2.5–14), and the median body weight was 29 kg (range, 4–60). Breeds included German Shepherd dogs (6/44, 13.6%), mixed‐breed dogs (11/44, 25.0%), Boxers (3/44, 6.8%), 2 dogs each (2/44, 4.5%) of American Staffordshire Terrier, Golden Retriever, Husky, and Yorkshire Terrier, and one each of Cocker Spaniel, Labrador Retriever, Dobermann Pinscher, English Pointer, Italian Hound, Samoyed, Giant Schnauzer, American Bulldog, Austrian Black and Tan Hound, Dog de Bordeaux, Epagneul Breton, Dachshund, Cavalier King Charles Spaniel, Cane Corso, Beauceron, and Bracco Italiano.

Seven of the 44 dogs were presented with extraparenchymal cyst recurrence after previous treatment. More specifically, three dogs had a history of ultrasound‐guided drainage of the fluid content, three dogs had undergone alcoholization, and one dog had had marsupialization of the cyst.

### 3.2 Clinical findings

The dogs were presented most frequently for gastrointestinal signs (tenesmus in 22/44 dogs; 50%) and urological signs (dysuria in 18/44 dogs; 40.9%). On physical examination, the main abnormalities were abdominal enlargement (6/44 dogs, 13.6%), pain on abdominal palpation (5/44 dogs, 11.4%), and swelling of the perineum (9/44 dogs, 20.5%). Abdominal and/or rectal palpation revealed either an enlarged prostate or an asymmetric, fluid‐filled structure in the caudal abdomen.

The comorbidities found during the physical examination were perineal hernia (in 10/44 dogs; 22.7%), testicular neoplasms (in 5/44 dogs; 11.4%; identified postoperatively as 2 seminomas, 2 Leydig cell tumors, and 1 Sertoli cell tumor), and inguinal hernia (in 1/44 dogs; 2.3%).

### 3.3 Clinicopathological data

The relevant preoperative blood abnormalities were azotemia in 3/44 dogs (6.8%), with increased serum urea (78, 87, and 103 mg/dl respectively; reference interval 21–60 mg/dl) and creatinine levels (2.7, 3.1, and 3.6 mg/dl, respectively; reference interval 0.5–1.4 mg/dl); serum potassium was 4.3, 4.7, and 5.2 mEq/L (range, 3.8–5.0 mEq/L), respectively. An indwelling urethral catheter was placed in the azotemic dogs, and fluid therapy was carried out in an attempt to stabilize them medically before surgery; however, azotemia persisted after medical management. Urinalysis was available in 23/44 dogs (52.3%); the results were unremarkable in 16 dogs, there was isosthenuria (urine specific gravity ranging from 1018 to 1014) in 4 dogs, while the remaining 3 dogs had signs of inflammation on microscopic sediment examination.

All the dogs underwent diagnostic imaging, consisting of abdominal ultrasound in the majority of cases (in 41/44 dogs; 93.2%). Other imaging modalities included abdominal radiographs (in 11/44 dogs; 25.0%), computed tomography (in 3/44 dogs; 6.8%), and excretory urography (in 2/44 dogs; 4.5%). Imaging revealed extraparenchymal cysts in 29 dogs (65.9%), intraparenchymal cysts in 11 dogs (25.0%) and concomitant intra‐ and extraparenchymal cysts in 4 dogs (9.1%). US‐recorded cyst dimensions were available in 23 dogs (17 extraparenchymal and 6 intraparenchymal). For dogs with extraparenchymal cyst, 9/17 (52.9%) had cysts measuring >20 cm and 8/17 (47.1%) had cysts ranging between 5 and 20 cm in diameter. In six dogs, the extraparenchymal cysts had both an abdominal and an intrapelvic extension while, in two dogs, the cysts were completely intrapelvic with perineal extension causing a perineal swelling. For dogs with intraparenchymal cysts, 3/6 (50.0%) had cysts between 5 and 20 cm, 2/6 (33.3%) <5 cm, and 1/6 (16.7%) had a cyst measuring >20 cm.

Signs of urinary tract obstruction were detected in 8/44 dogs (18.2%), including the 3 dogs previously reported with azotemia. Three dogs had bilateral hydronephrosis and hydroureters, two dogs had a severely distended bladder with inability to void, two dogs had a unilateral hydroureter, and one dog had a severely distended bladder and bilateral hydronephrosis. For the eight dogs with urinary tract obstruction, six had extraparenchymal cysts and two had intraparenchymal cysts.

### 3.4 Surgical findings and management

After urethral catheterization, the surgical procedure started with a prescrotal orchiectomy, except for the two unilateral cryptorchid dogs; in the latter, the retained abdominal testis was removed during the abdominal approach. In 43 dogs a caudal midline celiotomy was performed; in 4/43 dogs (9.3%), the extraparenchymal cysts required a combined caudal abdominal and lateral perineal approach. In one dog, the extraparenchymal cyst was treated by means of a lateral perineal approach only, and the cyst was completely resected.

For the extraparenchymal cysts (including the 4 dogs with both types of cysts, since the extraparenchymal cyst was interpreted as clinically more relevant), the procedure consisted of partial resection of the cyst and omentalization in 26/33 dogs (78.8%) and complete cyst removal in the remaining 7/33 dogs (21.2%). For all the intraparenchymal cysts (11 dogs), drainage and intracapsular omentalization was performed. In four dogs with both intra‐ and extraparenchymal cysts, omentalization of both types of cyst was performed.

Additional surgical procedures were performed in 9/44 dogs (20.5%), including perineal hernia repair (7 dogs), inguinal hernia repair (1 dog), and splenectomy (1 dog). In three dogs, a perineal herniorrhaphy was performed during a later surgical session. Postoperative antimicrobial treatment was used in 37/44 dogs (84.1%).

### 3.5 Intraoperative complications

In one dog with combined extra‐ and intraparenchymal cyst treatment, urine leakage from the prostatic urethra was noted before intracapsular omentalization (Table 1). The site of urethral leakage was closed with single interrupted sutures using 5/0 polydioxanone (PDS II, Ethicon, Guaynabo, Puerto Rico), and reinforced with omentum (which also served to drain the intraparenchymal cyst). After reconstruction, the operative urethral catheter was changed for a Foley catheter left in place until the death of the dog 2 days postoperatively.

**TABLE 1:** Type of surgical treatment, intra‐ and postoperative complications, recurrence, and outcome in the 44 dogs with sterile PCs included in the study

Lesion	Type of surgery	*N* cases	Intraoperative complication	Postoperative minor complication	Postoperative major complication	Recurrence	Death
Extra‐parenchymal cyst	CR	7	0	1 permanent urinary incontinence	0	0	0
	PRO	22	0	1 transient urinary retention, limb edema, pyrexia 1 transient urinary incontinence 3 permanent low‐grade urinary incontinence 1 dysuria	1 urinary retention + persistent hydronephrosis + cyst recurrence + acute kidney injury 1 cardiac arrhythmia	2	3
Combined intra and extra‐parenchymal cyst	PRO + DIO	4	1 urethral tear	0	1 acute kidney injury	0	1
Intra‐parenchymal cyst	DIO	11	0	1 transient urinary incontinence 1 permanent urinary incontinence 1 transient urinary retention	0	0	0

Abbreviations: CR, complete resection; DIO, drainage and intracapsular omentalization; *N*, number; PRO, partial resection and omentalization.

### 3.6 Postoperative outcomes

The median long‐term follow‐up for the 41 dogs surviving more than 6 months after surgery was 528 days (range, 215–730).

Major postoperative complications resulting in the death of the patient were observed in 3/44 dogs (6.8%), all of which had urinary tract obstruction preoperatively. Two dogs died 2 days after surgery, one dog (with intraoperative complication) due to acute kidney injury (AKI), the second dog due to cardiac arrhythmia, and the third dog died after 20 days because of persistence of urinary tract obstruction, urinary retention after removal of the urinary catheter, and recurrence of the extraparenchymal cyst. In the long‐term, one dog was euthanized after 18 months for urinary and fecal tenesmus presumably caused by PC recurrence (further investigation was declined by the owner).

Minor complications were observed in 10/44 dogs (22.7%) and consisted of urinary incontinence in 7 dogs (7/44, 15.9%) and transient urinary tract obstruction in 3 dogs (3/44, 6.8%). Urinary incontinence was permanent in 5/44 dogs (11.3%) and transient in 2/44 dogs (4.5%). One dog with permanent urinary incontinence was successfully treated with phenylpropanolamine (1 mg/kg every 8 h orally, Vetoquinol Italia srl, Bertinoro, Italy), in the remaining 4 dogs the urinary incontinence was low‐grade and it was not treated. Transient urinary tract obstruction consisted of transient urinary retention in 2/44 dogs (4.5%) and dysuria in 1/44 (2.3%). In one of the two dogs with urinary retention, transient hind limb edema and hyperthermia were also observed.

Recurrence of PCs was observed in 2/44 dogs (4.5%), both with extraparenchymal cysts treated by partial cyst removal and omentalization, and suspected in a third dog, also with partial cyst removal and omentalization. The times to recurrence were 20, 240, and 540 days. One dog was euthanized due to concomitant presence of urinary tract obstruction and azotemia, the second was managed with twice monthly percutaneous cyst drainage and the third dog was euthanized without further investigations. No recurrence occurred in dogs with extraparenchymal cysts when the cyst was completely removed (7 dogs) or dogs with intraparenchymal cysts (11 dogs).

Long‐term remission of clinical signs was observed in 39/44 dogs (88.6%) that underwent surgery.

## 4 DISCUSSION

Surgery for the treatment of PCs was successful in the long term in 88.6% (39/44) of the dogs. Success was observed in 21/26 dogs (80.8%) with extraparenchymal cyst and in all the dogs with intraparenchymal cysts. To the best of the authors' knowledge, the present study represents the largest case series regarding the treatment of sterile PCs.

For extraparenchymal cysts, the choice between a complete resection or partial resection and omentalization was performed according to the amount of adhesion present between the cyst and the surrounding organs. If the adhesion were few and easy to dissect, complete resection was preferred.

The only intraoperative complication reported was the occurrence of urine leakage from the prostatic urethra in one dog. This major intraoperative complication was resolved during surgery and no evidence of urine leakage was observed in the postoperative abdominal ultrasound examination. It is unclear if the urethral wall ruptured during the surgical procedure or an abnormal communication between the urethra and the cyst was already present, as has already been reported in the literature,^7^, ^8^ which was then enlarged during the surgical maneuvers. The urethral defect was sutured, and a Foley catheter placed,^8^ but the dog died after 2 days postoperatively due to AKI. Preoperatively, the dog had signs of urinary tract obstruction and azotemia which could have predisposed to AKI after surgery. It is the authors' opinion that the intraoperative use of a semi‐rigid urinary catheter during prostatic surgery, instead of a soft catheter such as a Foley catheter, greatly helps with the identification of the urethra during intracapsular omentalization, where there is a risk of iatrogenic damage of the urethra. In the case of cystic lesions close to the urethra or involving the peri‐urethral tissues, intraoperative ultrasound might be useful in guiding the surgeon in the dissection. Natural openings in the prostatic urethra need to be differentiated from iatrogenic urethral tears. Retracting the semi‐rigid catheter allows to perform urethral flushing, thus checking for urethral integrity. Reconstruction of the urethra is possible for visible defects, but the application of an adequate amount of omentum may be sufficient to seal small openings.^8^

A total of four dogs died of short and long‐term postoperative complications. Three dogs experienced postoperative AKI. They all presented with urinary tract obstruction and 2/3 dogs (66.7%) had preoperative azotemia. The urinary tract obstruction was considered to have occurred due to PC compression. Surgical treatment did not resolve the urinary obstruction leading to further deterioration of renal function. Possible causes of AKI were sustained inflammation induced by surgery which prevented resolution of the obstruction, chronic anatomical and functional changes, or possible hypotension during surgery. Although unlikely, underlying chronic kidney disease decompensated postoperatively by the urinary tract obstruction cannot be completely excluded in these cases. The fourth dog was euthanized after 18 months for clinical signs linked to suspected cyst recurrence.

Minor postoperative complications were observed in 10 dogs and consisted mainly of urinary incontinence and urinary retention. Incontinence was temporary in two dogs, while in four dogs, the acquired urinary incontinence was low‐grade but permanent and no treatment was undertaken; in the fifth dog, medical treatment was successful. Urinary retention or incontinence have already been reported as the most frequent complications after prostatic surgery^19^ and can be caused by multiple factors. Large cysts can severely alter the anatomy of both the prostate and bladder trigone and neck, inducing chronic changes in the neurological control of voiding.^17^ Surgery can cause innervation damage, especially if the dissection is performed along the dorsal and lateral aspect of the bladder, prostate, and urethra.^17^ If partial cyst removal and omentalization are performed, these areas should be spared, thus decreasing the chances of neurological damage.^19^ Intracapsular omentalization of the intraparenchymal cysts was performed when feasible, according to the modification proposed by Zambelli and Bralia^20^ in which a ventral opening of the prostate was suggested in an attempt to avoid the neurovascular structures.

Castration has historically been considered a risk factor for the development of urinary incontinence in male dogs.^21^, ^22^ However, its role in the development of urinary incontinence has recently been questioned since Hall et al.^23^ did not report an increased prevalence of incontinence in castrated male dogs. In case of permanent urinary incontinence, ruling out urinary infections or pelvic bladder position as causes, the latter being possible after resolution of PCs is imperative.^22^ Medical treatment includes the use of phenylpropanolamine; however, in males the success rate of the medical treatment for urinary incontinence is usually lower in comparison to females.^24^ In refractory cases, the placement of a hydraulic urethral occluder has been suggested.^24^ Despite urinary incontinence being considered a minor complication, refractory incontinence may necessitate additional medical interventions, and can negatively influence the owner–dog relationship warranting proper owner counseling before treatment of PC.^25^

The recurrence of extraparenchymal cysts was observed in 2/44 dogs (4.5%) treated by partial resection and omentalization and suspected in a third dog also treated with the same procedure. The recurrence rate after surgery for PCs has been reported to range from 0% to 33%^10^, ^17^; however, few studies report the long‐term postoperative outcome.^10^, ^16^ The reasons for recurrence may be a failure to remove all the septa in the case of multilobulated cysts or to omentalize all the regions of the cyst during surgery. Moreover, castration may help to prevent the recurrence of intraparenchymal cysts much more effectively than the recurrence of extraparenchymal cysts. In the case of recurrence of a PC, surgical revision or minimally invasive procedures may be applied.

In the present study, castration was part of the standard treatment. In the literature, it has been suggested for all PCs, without distinction between intra‐ and extraparenchymal cysts.^5^, ^8^ The former is frequently associated with BPH. Castration eliminates the hormonal influence on the prostate and would therefore be indicated for intraparenchymal cysts.^5^, ^19^ On the contrary, castration for extraparenchymal cysts is less justifiable. In fact, the latter is external to the prostate gland and usually connected only to the prostatic capsule and no hormonal influence has been demonstrated regarding their development. Extraparenchymal cysts have also been reported in castrated dogs.^18^ The common practice of castrating dogs even in cases of extraparenchymal cysts may be just routine, but, in part, it may be related to concomitant diseases requiring castration (cryptorchidism, testicular tumor, perineal hernia, BPH, and prostatitis). Additional studies are needed to understand whether castration is really necessary for treating extraparenchymal cysts.

The median age and body weight are in line with previous data.^2^, ^3^, ^8^, ^9^, ^17^ Mixed breed dogs, German Shepherd dogs, and Boxers were the most affected breeds in this study. Although no clear breed predisposition has been reported in the literature,^5^ the study by Bray et al.^17^ also found Boxers to be overrepresented.

No clear guidelines are available in the literature regarding patient selection for either minimally invasive procedures^8^, ^12^, ^13^, ^14^ or open surgery. It is likely that the size of the cyst could influence the choice of the procedure. It is important to note that ultrasound‐guided drainage may help to identify sterile versus infected cysts,^8^ and possibly to stabilize patients before surgery. In particular, in the case of urinary tract obstruction and azotemia PC drainage could, at least temporarily, alleviate the urethral obstruction.

The main limitation of this study is its retrospective nature, with some of the data missing (e.g., cyst dimensions were only available for 23 dogs); in addition, creatinine was not evaluated in the fluid drained. The other important limitation is the subjectivity related to the surgical technique selected by the surgeon and the low number of cases for some of the surgical procedures used. Moreover, it would have been interesting to follow these dogs postoperatively with serial abdominal ultrasound examinations in order to evaluate whether the lack of clinical signs corresponded to real resolution.

In conclusion, surgery for sterile PCs is effective and safe. The most frequent postoperative complication observed in this retrospective study was urinary incontinence. Nevertheless, lethal postoperative complications, from persistent urinary tract obstruction and/or progressive kidney damage, were infrequently encountered and should be discussed with the owner in advance.

## CONFLICT OF INTEREST

The authors declare no conflicts of interest related to this report.

## AUTHOR CONTRIBUTIONS

Sara Del Magno analyzed the data, wrote and revised the paper, and contributed to the surgeries performed at Ospedale Universitario Veterinario, University of Turin (two cases). Guido Pisani and Filippo Cinti performed the surgeries at the Centro Veterinario Luni Mare (12 cases) and took part in the design of the study. Francesco Dondi performed the statistical analysis, helped in the analysis of data, writing and revising the manuscript. Emanuela Morello helped in collecting the data, writing the article and revision, and contributed with some of the cases at the Ospedale Universitario Veterinario, University of Turin (six cases). Marina Martano helped in collecting data, writing the article and revision, and contributed with cases at the Ospedale Universitario Veterinario, University of Turin (six cases). Armando Foglia helped in the analysis of the data and the revision of the manuscript. Davide Giacobino helped in the management of cases at the Ospedale Universitario Veterinario, University of Turin, helped in the collection of data and revision of the manuscript. Paolo Buracco was responsible for the study design, helped in writing and revising the manuscript, and contributed with the cases at the Ospedale Universitario Veterinario, University of Turin (18 cases).

## Data Availability

The data that support the findings of this study are available upon request from the corresponding author. The data are not publicly available due to privacy or ethical restrictions.
